# Genetic diversity and population structure of the tsetse fly *Glossina fuscipes fuscipes* (Diptera: Glossinidae) in Northern Uganda: Implications for vector control

**DOI:** 10.1371/journal.pntd.0005485

**Published:** 2017-04-28

**Authors:** Robert Opiro, Norah P. Saarman, Richard Echodu, Elizabeth A. Opiyo, Kirstin Dion, Alexis Halyard, Augustine W. Dunn, Serap Aksoy, Adalgisa Caccone

**Affiliations:** 1 Department of Biology, Faculty of Science, Gulu University, Gulu, Uganda; 2 Department of Ecology and Evolutionary Biology, Yale University, New Haven, Connecticut, United States of America; 3 Division of Genetics and Genomics, Boston Children's Hospital, Boston, Massachusetts, United States of America; 4 Department of Epidemiology of Microbial Diseases, Yale School of Public Health, New Haven, Connecticut, United States of America; Institut de recherche pour le developpement, FRANCE

## Abstract

Uganda is the only country where the chronic and acute forms of human African Trypanosomiasis (HAT) or sleeping sickness both occur and are separated by < 100 km in areas north of Lake Kyoga. In Uganda, *Glossina fuscipes fuscipes* is the main vector of the *Trypanosoma* parasites responsible for these diseases as well for the animal African Trypanosomiasis (AAT), or Nagana. We used highly polymorphic microsatellite loci and a mitochondrial DNA (mtDNA) marker to provide fine scale spatial resolution of genetic structure of *G*. *f*. *fuscipes* from 42 sampling sites from the northern region of Uganda where a merger of the two disease belts is feared. Based on microsatellite analyses, we found that *G*. *f*. *fuscipes* in northern Uganda are structured into three distinct genetic clusters with varying degrees of interconnectivity among them. Based on genetic assignment and spatial location, we grouped the sampling sites into four genetic units corresponding to northwestern Uganda in the Albert Nile drainage, northeastern Uganda in the Lake Kyoga drainage, western Uganda in the Victoria Nile drainage, and a transition zone between the two northern genetic clusters characterized by high level of genetic admixture. An analysis using *HYBRIDLAB* supported a *hybrid swarm* model as most consistent with tsetse genotypes in these admixed samples. Results of mtDNA analyses revealed the presence of 30 haplotypes representing three main haplogroups, whose location broadly overlaps with the microsatellite defined clusters. Migration analyses based on microsatellites point to moderate migration among the northern units located in the Albert Nile, Achwa River, Okole River, and Lake Kyoga drainages, but not between the northern units and the Victoria Nile drainage in the west. Effective population size estimates were variable with low to moderate sizes in most populations and with evidence of recent population bottlenecks, especially in the northeast unit of the Lake Kyoga drainage. Our microsatellite and mtDNA based analyses indicate that *G*. *f*. *fuscipes* movement along the Achwa and Okole rivers may facilitate northwest expansion of the Rhodesiense disease belt in Uganda. We identified tsetse migration corridors and recommend a rolling carpet approach from south of Lake Kyoga northward to minimize disease dispersal and prevent vector re-colonization. Additionally, our findings highlight the need for continuing tsetse monitoring efforts during and after control.

## Author summary

Northern Uganda is an epidemiologically important region affected by human African trypanosomiasis (HAT) because it harbors both forms of the HAT disease (*T*. *b*. *gambiense* and *T*. *b*. *rhodesiense*). The geographic location of this region creates the risk that these distinct foci could merge, which would complicate diagnosis and treatment, and may result in recombination between the two parasite strains with as yet unknown consequences. Both strains require a tsetse fly vector for transmission, and in Uganda, *G*. *f*. *fuscipes* is the major vector of HAT. Controlling the vector remains one of the most effective strategies for controlling trypanosome parasites. However, vector control efforts may not be sustainable in terms of long term reduction in *G*. *f*. *fuscipes* populations due to population rebounds. Population genetics data can allow us to determine the likely source of population rebounds and to establish a more robust control strategy. In this study, we build on a previous broad spatial survey of *G*. *f*. *fuscipes* genetic structure in Uganda by adding more than 30 novel sampling sites that are strategically spaced across a region of northern Uganda that, for historical and political reasons, was severely understudied and faces particularly high disease risk. We identify natural population breaks, migration corridors and a hybrid zone with evidence of free interbreeding of *G*. *f*. *fuscipes* across the geographic region that spans the two HAT disease foci. We also find evidence of low effective population sizes and population bottlenecks in some areas that have been subjects of past control but remain regions of high tsetse density, which stresses the risk of population rebounds if monitoring is not explicitly incorporated into the control strategy. We use these results to make suggestions that will enhance the design and implementation of control activities in northern Uganda.

## Introduction

The tsetse fly (genus *Glossina*) is the major vector of human African trypanosomiasis (HAT) and animal African trypanosomiasis (AAT). The diseases occur throughout sub-Saharan Africa, causing extensive morbidity and mortality in humans and livestock [1][2]. Human disease is caused by two different subspecies of the flagellated protozoa *Trypanosoma brucei*; *T*. *b*. *rhodesiense* in eastern and southern Africa, and *T*. *b*. *gambiense* in west and central Africa. The two HAT diseases are separated geographically more or less along the line of the Great Rift Valley [3]. Although the animal disease (or Nagana) is caused by different trypanosome subspecies; *T*. *b*. *brucei*, *T*. *congolense* and *T*. *vivax*, animals are also known to be reservoirs of the human infective *T*. *b*. *rhodesiense*. Thus, while AAT is a problem in its own right because of economic losses and reduced availability of nutrients [4][5][6], the animals also act as important reservoirs for human infective *T*. *b*. *rhodesiense*.

Although the human diseases have been on a decline[7], they still put 60 million people at risk in 37 countries covering about 40% of Africa [8][9]. The human disease *T*. *b*. *gambiense* is near elimination while control of *T*. *b*. *rhodesiense* remains more complicated because of animal reservoirs. For both *T*. *b*. *gambiense* and *T*. *b*. *rhodesiense*, there are no prophylactic drugs or vaccines available. Furthermore, the drugs for treatment are expensive, can cause severe side effects, and are difficult to administer in remote villages [10][11]. Although AAT can be prevented with prophylactic drugs and effectively treated with trypanocidal drugs, progress towards elimination of the animal disease has been slow because of the high cost of drug administration and repeated emergence of drug resistance [12]. Thus, AAT instances remain high and continue to burden livestock farmers [13] and provide animal reservoirs of *T*. *b*. *rhodesiense*. As a consequence, the most effective way to control both AAT and HAT is control of the tsetse vector [14].

Uganda is in the precarious position of being the only country that harbors both forms of HAT, with *T*. *b*. *gambiense* present in the northwestern corner of the country and *T*. *b*. *rhodesiense* found in the center and southeast [15]. There is a significant risk that the two sleeping sickness subspecies will merge in the north-central districts of Uganda, a region already burdened by political and social instability [16]. Merging of the two disease belts would complicate treatment and diagnosis [17], and may lead to the emergence of unforeseen pathologies if there is recombination between the *T*. *b*. *gambiense* and *T*. *b*. *rhodesiense* trypanosomes [18][19][20].

**Fig 1:**
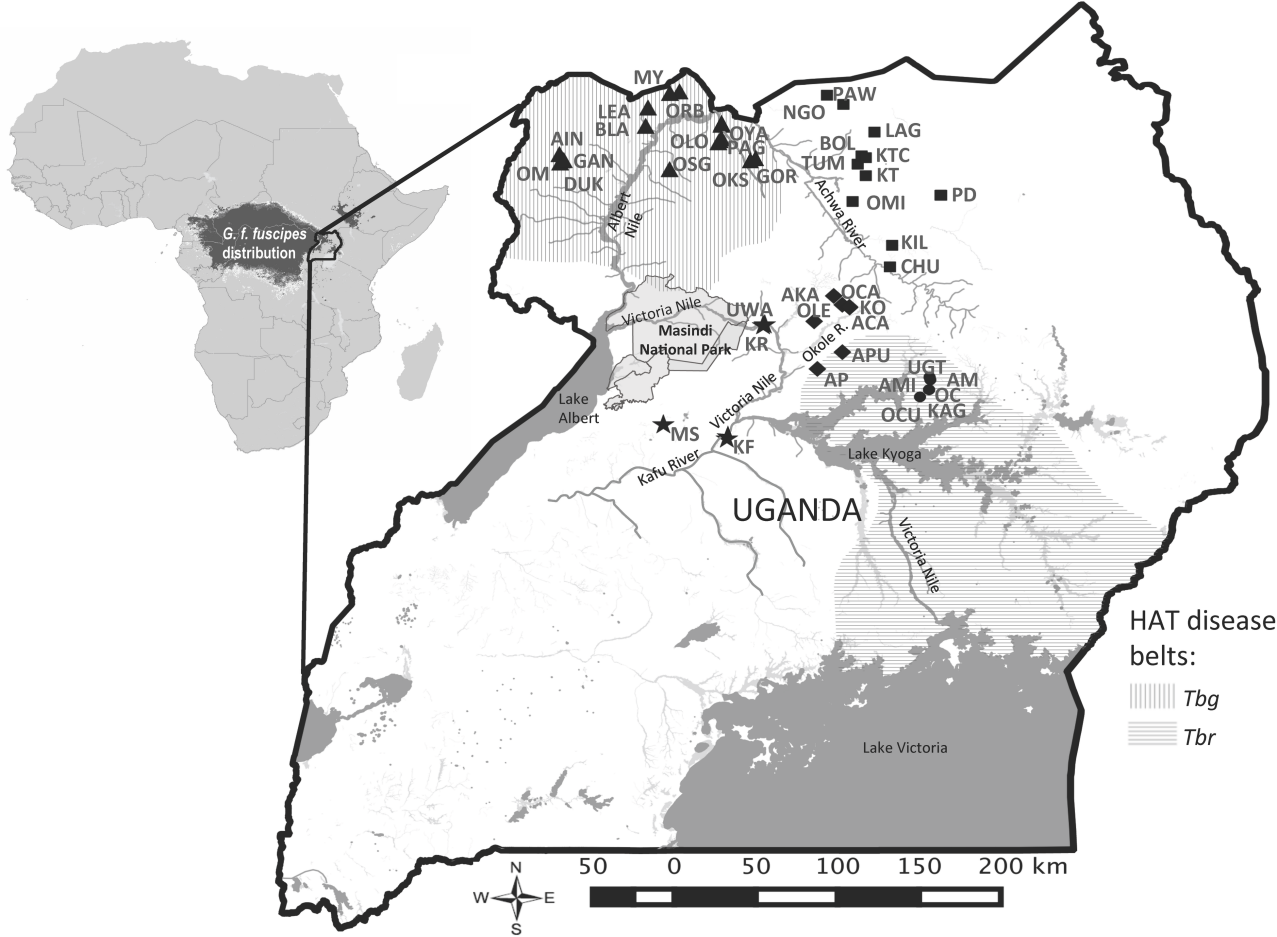
Map of Uganda showing sampling area. Markers indicate sampling sites for the major drainage basins; Albert Nile sites are marked with triangles, Achwa River sites are marked with squares, Okole River sites are marked with diamonds, Lake Kyoga sites are marked with circles, and Victoria Nile sites are marked with stars (see Table 1 for details). Vertical and horizontal stripes indicate the approximate distribution of the two *Trypanosoma* (*T*. *brucei gambiense* [*Tbg*] and *T*. *brucei rhodesiense* [*Tbr*]) responsible for the two sleeping sickness disease forms in Uganda. The major water bodies (lakes and rivers) are identified with a dark shade of gray.

*Glossina fuscipes fuscipes* is a member of the *palpalis* group of tsetse and is the main vector implicated in the transmission of both AAT and HAT in Uganda. The vector is distributed over vast regions of sub-Saharan Africa (Fig 1), where it occupies discrete patches of riverine and lacustrine habitats distributed among pasture and agricultural land. Assessing the population structure and the extent to which these apparently discrete populations are connected by dispersal and migration patterns is central to defining the most effective scale for vector control [21][22]. For example, the major challenge that faces most control efforts is tsetse rebound following short-term control efforts. The source of rebounding populations could be residual pockets of surviving individuals or migrant flies coming from neighboring untreated regions, or both [23]. Increased knowledge of vector population dynamics through application of population genetics can help in assessing the suitability of the operational units selected for vector control and result in more effective tsetse control.

Although previous studies have made great strides towards understanding the population biology of *G*. *f*. *fuscipes* in Uganda [24][25][26][27][28], regions north of Lake Kyoga remain a high priority for additional study. Northern Uganda harbors both forms of HAT in close geographic proximity [7][20]. Identifying the precise extent of the two disease belts and possible risk of merger has been difficult until recently because of social and political upheaval experienced in these regions [29][30]. Our previous population genetic studies have identified three major genetic units present in north and south of Lake Kyoga and in western Uganda [24][25][31]. Each of these units consists of genetically distinct populations with high differentiation between sampling sites and evidence of further sub-structuring [22][25][31][24].

A more detailed understanding of the genetic structure and population dynamics of *G*. *f*. *fuscipes* in northern Uganda will help estimate the likelihood of the merger of the two HAT disease forms, and identify the best tsetse control strategies for the region. In this study, we comprehensively sampled *G*. *f*. *fuscipes* from 42 sites in areas north of Lake Kyoga and assessed variation in 16 nuclear microsatellites and over a 570 bp region of mitochondrial DNA (mtDNA) to understand both short time scale resolution of demographic events [32][33] and inference of phylogeographic events dating further back in time [34][35]. We compared this new knowledge of fine scale population structure, migration patterns and population dynamics in northern Uganda with previous studies that concentrated on the southern and central regions of the country. This comparative approach allowed us deeper insights into the evolutionary forces at play and enriched our ability to make recommendations for *G*. *f*. *fuscipes* control strategies in northern Uganda.

## Materials and methods

### Study area and tsetse samples

The map in Fig 1 shows the sampling sites. We used biconical traps [36] to collect from 30 sites between January 2014 and April 2015, and also included 12 collections from a previous study between January 2008 and January 2012 [31][25]. Sampling sites were chosen to detect fine spatial scale population structure. To do this, we collected from multiple sites separated by just over 5 km, which is the smallest unit area for which genetic differentiation has been observed in *G*. *f*. *fuscipes* in Uganda [25]. At each site, we placed an average of 6 traps at least 100 m from each other and collected an average of 18 flies per trap over a period of 3–4 days. Flies were stored individually in 95% ethanol and information on sex, collection date, trap number, and geographic coordinates of each trap was recorded. The genotypic data included from previous studies [25][29] were separated from our samples by a time span of 3–7 years (approx. 22–52 generations), which opened up the possibility of genetic change. However, a previous study showed no evidence of large demographic changes between the temporal collections [37], justifying the combined analyses of 42 sampling sites spanning these time points (details in S1 Table).

We extensively sampled in areas north of Lake Kyoga, which includes tsetse flies that in a previous study were grouped into two genetic clusters [25], with an effort to sample major water drainage systems. [25] described one genetic cluster north of the Lake Kyoga and the Victoria Nile, and one in western Uganda. In the northern genetic cluster, we sampled the Albert Nile, the Achwa River, the Okole River, and Lake Kyoga drainages (see Table 1). The Albert Nile basin is in the far northwest corner of Uganda, a region known to be an active focus of *T*. *b*. *gambiense* sleeping sickness [20][38]. The Albert Nile is bordered to the east and eventually joined by the Achwa River, and both drainage systems generally consist of patchy habitat suitable for *G*. *f*. *fuscipes*, characterized by lowland woodland near semi-permanent water bodies [2]. Habitat patches are surrounded by unsuitable savannah, agricultural and pastoral lands. Although the district of Arua was recently included in a pilot vector control program in 2011–2013 [[39]], our samples from 2014 (DUK, AIN and GAN) that may have been impacted did not overlap spatially with the program. Further south, we sampled the Okole River and Lake Kyoga basins (Fig 1, Table 1). These regions form vast areas of marsh and swampland, and are the most northerly geographical extent of the *T*. *b*. *rhodesiense* HAT disease belt [40]. Some districts in the Lake Kyoga drainage, such as Dokolo and K’maido, were targets of the Stamp Out Sleeping Sickness (SOS) campaign of 2006–2009 [41], which may have impacted our samples from this region from 2009 (OC) and 2014 (OCU, AMI and KAG). Finally, in the western genetic cluster described by [25], we collected samples along and south of the Victoria Nile (Fig 1, Table 1), which flows northwest from Lake Kyoga into Lake Albert on the edge of the western rift valley. Here we sampled sites along and on minor tributaries of the Victoria Nile in the districts of Masindi and Kiryandongo (Table 1). This region is characterized by lowland woodland and the Uganda Wildlife Authority protects much of the region as part of the Murchison Falls National Park.

**Table 1:** Sampling localities and microsatellite and mtDNA assignment.

General information					Microsatellites			mtDNA					Genetic unit assignment
Population	Closest Village	District	Drainage Basin	N	Cluster-1 average q	Cluster-2 average q	Cluster-3 average q	N sequences	Count A	Count B	Count C	% mismatched	
**Northwest: Overall statistics**				**311**	**0.90**	**0.05**	**0.04**	**173**	**130**	**43**	**0**	**26.2%**	**Northwest**
DUK	Duku	Arua	Albert N.	25	0.89	0.04	0.07	13	13	0	0	0.0%	Northwest
AIN	Aina	Arua	Albert N.	19	0.91	0.04	0.05	10	9	1	0	0.0%	Northwest
GAN	Gangu	Arua	Albert N.	20	0.88	0.06	0.06	11	11	0	0	0.0%	Northwest
*OM	Omugo	Arua	Albert N.	15	0.87	0.07	0.06	15	15	0	0	0.0%	Northwest
OSG	Osugo	Moyo	Albert N.	20	0.84	0.06	0.09	12	12	0	0	0.0%	Northwest
BLA	Belameling	Moyo	Albert N.	10	0.93	0.05	0.02	9	8	1	0	11.1%	Northwest
LEA	Lea	Moyo	Albert N.	8	0.91	0.05	0.04	6	3	3	0	50.0%	Northwest
ORB	Orubakulemi	Moyo	Albert N.	20	0.93	0.03	0.04	11	8	3	0	27.3%	Northwest
*MY	Moyo	Adjumani	Albert N.	15	0.87	0.08	0.05	17	15	2	0	16.7%	Northwest
OLO	Olobo	Adjumani	Albert N.	24	0.88	0.07	0.05	10	7	3	0	20.0%	Northwest
OYA	Oringya	Adjumani	Albert N.	9	0.94	0.02	0.04	8	6	2	0	25.0%	Northwest
PAG	Pagirinya	Adjumani	Albert N.	20	0.92	0.04	0.04	9	5	4	0	44.4%	Northwest
OKS	Okidi	Amuru	Albert N.	26	0.83	0.15	0.02	11	8	3	0	27.3%	Northwest
GOR	Gorodona	Amuru	Albert N.	25	0.89	0.09	0.02	12	8	4	0	16.7%	Northwest
NGO	Ngomoromo	Lamwo	Achwa R.	25	0.96	0.02	0.02	10	2	8	0	80.0%	Northwest
PAW	Pawor	Lamwo	Achwa R.	13	0.95	0.02	0.03	0	0	0	0	N/A	Northwest
LAG	Lagwel	Lamwo	Achwa R.	17	0.95	0.03	0.03	9	0	9	0	100%	Northwest
**Transition Zone: Overall statistics **				**310**	**0.54**	**0.43**	**0.03**	**150**	**36**	**114**	**0**	**19.9%**	**Transition**
BOL	Bola	Kitgum	Achwa R.	25	0.80	0.18	0.01	10	3	7	0	10.0%	Transition
TUM	Tumangu	Kitgum	Achwa R.	20	0.76	0.21	0.03	10	3	7	0	10.0%	Transition
KTC	Kitgum	Kitgum	Achwa R.	20	0.76	0.22	0.02	9	1	8	0	33.3%	Transition
*KT	Kitgum	Kitgum	Achwa R.	17	0.87	0.08	0.04	9	2	7	0	55.6%	Transition
OMI	Omido	Pader	Achwa R.	15	0.66	0.32	0.02	9	2	7	0	22.2%	Transition
*PD	Pader	Pader	Achwa R.	13	0.63	0.34	0.03	10	1	9	0	30.0%	Transition
KIL	Kilak	Pader	Achwa R.	21	0.37	0.56	0.06	9	2	7	0	22.2%	Transition
CHU	Chua	Pader	Achwa R.	25	0.25	0.73	0.03	9	5	4	0	11.1%	Transition
OCA	Ocala	Oyam	Okole R.	20	0.51	0.46	0.03	9	3	6	0	22.2%	Transition
AKA	Akayo-debe	Oyam	Okole R.	26	0.39	0.57	0.04	9	1	8	0	11.1%	Transition
*KO	Kole	Oyam	Okole R.	15	0.45	0.52	0.03	9	2	7	0	22.2%	Transition
OLE	Olepo	Kole	Okole R.	24	0.37	0.61	0.02	9	3	6	0	11.1%	Transition
ACA	Acanikoma	Kole	Okole R.	25	0.64	0.31	0.06	11	3	8	0	9.1%	Transition
APU	Aputu-Lwaa	Apac	Okole R.	29	0.19	0.79	0.02	13	1	12	0	7.7%	Transition
*AP	Apac	Apac	Okole R.	15	0.44	0.53	0.04	15	4	11	0	20.0%	Transition
**Northeast: Overall statistics**				**184**	**0.03**	**0.95**	**0.01**	**80**	**3**	**77**	**0**	**2.9%**	**Northeast**
*UGT	Kaberamaido	Dokolo	L. Kyoga	64	0.02	0.97	0.01	20	2	18	0	10.0%	Northeast
*AM	Aminakwach	Dokolo	L. Kyoga	30	0.02	0.97	0.01	16	0	16	0	0.0%	Northeast
AMI	Aminakwach	Dokolo	L. Kyoga	25	0.02	0.98	0.01	10	0	10	0	0.0%	Northeast
*OC	Oculoi	K’maido	L. Kyoga	20	0.05	0.94	0.02	14	1	13	0	7.1%	Northeast
OCU	Oculoi	K'maido	L. Kyoga	25	0.04	0.95	0.01	11	0	11	0	0.0%	Northeast
KAG	Kangai	K'maido	L. Kyoga	20	0.07	0.92	0.01	9	0	9	0	0.0%	Northeast
**West: Overall statistics**				**149**	**0.02**	**0.02**	**0.96**	**78**	**58**	**0**	**20**	**N/A**	**West**
UWA	Uganda WA	K'dongo	Victoria N.	25	0.02	0.01	0.97	11	7	0	4	N/A	West
*KR	Karuma	K'dongo	Victoria N.	60	0.04	0.02	0.94	9	7	0	2	N/A	West
*KF	Kafu	K'dongo	Victoria N.	34	0.01	0.01	0.98	3	3	0	0	N/A	West
*MS	Masindi	Masindi	Victoria N.	30	0.02	0.02	0.96	55	41	0	14	N/A	West

Sampling localities ordered from north to south: General information includes population, closest village, district, drainage basin, and number of samples included (N). Microsatellite results include average probability of assignment (q-value) to STRUCTURE defined clusters (1–3) based on 16 microsatellites. mtDNA results include number of sequences analyzed (N sequences), counts of each mtDNA haplogroup (A-C) and % mismatch with Microsatellite cluster assignment on an individual basis. The last column indicates genetic unit assignment. Genetic units were assigned based on each populations' mean STRUCTURE assignment; populations with >0.8 average q were assigned to pure genetic units (Northwest, Northeast, and West), and populations with <0.8 average q were assigned to the Transition Zone.

* indicates samples collected prior to 2014.

### DNA extraction and microsatellite genotyping

DNA was extracted from two to three legs per sample, using PrepGEM Insect DNA Extraction kit (ZyGEM New Zealand, 2013), following the manufacturer’s protocols and stored at -20°C. We collected genotypic data from 16 microsatellite loci (details in S2 Table). Amplifications were performed with fluorescently labeled forward primers (6-FAM, HEX and NED) using a touchdown PCR (10 cycles of annealing at progressively lower temperatures from 60°C to 51°C, followed by 35 cycles at 50°C) in 13.0μl reaction volumes containing 2.6 μl of 5X PCR buffer, 1.1 μl of 10 mM dNTPs, 1.1 μl of 25mM MgCl_2_ and 0.1 μl of 5 units/μl GoTaq (Promega, USA), 0.1 μl of 100X BSA (New England Biolabs, USA), 0.5 μl of 10 μM fluorescently-labeled M13 primer, 0.5 of μl 10 μM reverse primer, and 0.3 μl of 2 μM M13-tailed forward primer. For loci C7b and GmL11, 0.5 units of Taq Gold polymerase (Life Technologies, USA) were used instead of Promega GoTaq. PCR products were multiplexed in groups of two or three and genotyped on an ABI 3730xL Automated Sequencer (Life Technologies, USA) at the DNA Analysis Facility on Science Hill at Yale University (http://dna-analysis.yale.edu/). Alleles were scored using the program GENEMARKER v2.4.0 (Soft Genetics, State College, PA, USA) with manual editing of the automatically scored peaks.

### mtDNA amplification and sequencing

We followed the protocol designed by [25] to sequence a 570 bp fragment of mtDNA that spans the COI and COII genes. Briefly, we used primers COIF1 (5’–CCT CAA CAC TTT TTA GGT TTA G– 3’) and COIIR1 (5’–GGT TCT CTA ATT TCA TCA AGT A– 3’) to amplify 570 bp with an initial denaturation step at 95°C for 5 min, followed by 40 cycles of annealing at 50°C, and a final extension step at 72°C for 20 min. We used a reaction volume of 13.0 μl containing 1 μl of template genomic DNA, 2.6 μl of 5X PCR buffer, 1.1 μl of 10 mM dNTPs, 0.5 μl of 10mM primers, 1.1 μl of 25 mM MgCl2, and 0.1 μl (U/μL) of GoTaq polymerase (Promega, USA). The PCR products were purified using ExoSAP-IT (Affymetrix Inc., USA) as per the manufacturer’s protocol. Sequencing was carried out for both forward and reverse strands on the ABI 3730xL automated sequencer at the DNA Analysis Facility on Science Hill at Yale University (http://dna-analysis.research.yale.edu/). Sequence chromatograms were inspected by eye and sequences trimmed to remove poor quality data using GENEIOUS v6.1.8 (Biomatters, New Zealand). The forward and reverse strands were used to create a consensus sequence for each sample, and the sequences trimmed to a length of 490 bp. Only a subset of the samples screened for microsatellite variation was also sequenced at the mtDNA locus (Table 1).

### Microsatellite marker validation

For nuclear microsatellite marker validation, we tested for neutrality and independence with GENEPOP v4.2 [42]. We tested for departures from Hardy-Weinberg (HW) proportions in each sample and microsatellite locus using an approximation of an exact test based on a Markov chain iteration (10,000 dememorization steps, 1000 batches, 10,000 iterations per batch in the Markov chain); significance values were obtained following the Fisher’s method that combines probabilities of exact tests [43]. We tested for genotypic linkage disequilibrium (LD) among each pair of loci using the Guo and Thompson method [44]. To correct for false assignments of significance by chance alone for all simultaneous statistical tests and comparisons, we used the Benjamini-Hochberg False Discovery Rate (FDR) method [45]as opposed to the Bonferroni correction, because of lower incidence of false negatives[45][46].

### Microsatellite genetic diversity and population structure

For nuclear microsatellite data, we assigned individuals to genetic units without prior information on sampling locality with STRUCTURE v2.3.4 [47][48]. STRUCTURE simultaneously identifies unique genetic units and provides a probability of assignment (q-value, ranging from 0 to 1) for each individual. Twenty independent replicate runs for each K = 1–10 were carried out with an admixture model, independent allele frequencies, and a burn-in value of 50,000 steps followed by 250,000 iterations. The optimal value of K was determined using STRUCTURE HARVESTER v0.6 [49] to calculate the *ad hoc* statistic “ΔK” [50], and independent replicates were aligned with CLUMPP v1.1.2 [51].

In addition to STRUCTURE, we performed Discriminant Analysis of Principal Components (DAPC) with the "adegenet" package v1.4–2 [52] in the R version 3.0.2 environment [53]. The DAPC is a multivariate, model-free method that makes no assumptions about deviations from Hardy Weinberg and linkage disequilibrium, designed to describe patterns of genetic clustering among groups of individuals [54]. In this analysis, we grouped samples by their site of origin and used the cross-validation formula available to choose number of principal components (PCs) to retain. To understand the partitioning of microsatellite variance within and between genetic units, we performed an analysis of molecular variance (AMOVA) in ARLEQUIN v3.5 [55].

Genetic diversity indices including observed heterozygosity (H_O_), expected heterozygosity (H_E_), number of alleles, allelic richness (A_R_) and [56] estimator of inbreeding coefficient (F_IS_) were calculated in GENALEX v6.501 [57]. Pairwise differentiations at different hierarchical levels were estimated with two F-statistics. For comparison with previous *G*. *f*. *fuscipes* studies, we calculated Wright’s F-statistics [58], following the variance method developed by [56] using 10,000 permutations in ARLEQUIN. For accuracy with highly polymorphic markers [59], we estimated Jost’s D_EST_ with the R package DEMEtics [60][53], where p-values and confidence intervals were calculated based on 1000 bootstrap resamplings. With the resulting F-statistics, we tested for isolation by distance (IBD) using Rousset’s procedure [61] implemented in the “isolation by distance” v3.23 web service [62]. Geographic distances were generated using the web-based “geographic matrix generator” v1.2.3 [63]. The significance of the regression was tested by a Mantel test with 10,000 randomizations [64].

### Effective population size (Ne) and bottleneck analysis

Using microsatellite data, we estimated effective population size (Ne) for each sampling site independently. We did not group sites based on assignment to genetic clusters because strong evidence of substructure within clusters would violate assumptions. We estimated Ne using two methods implemented in N_E_ESTIMATOR v2.01 [65]: the modified two-sample temporal method [66] based on [67] for sites with multiple temporal samples, and the one-sample linkage disequilibrium (LD) method [68] for all 42 sites. We used two methods to estimate Ne because they each have different strengths and weaknesses [66,69–71]. The two-sample temporal method [64] is useful because it is robust when there are overlapping generations [71], but only provides an average estimate across two time points assuming a closed population, so cannot be used to assess the impact of control efforts. On the other hand, the LD method [66] is useful because it can provide an estimate for each sampling point and employs the bias corrections by [72], but is influenced by bias associated with non-overlapping generations and is not powerful enough to distinguish from infinite population sizes when there are insufficient polymorphisms and numbers of markers to detect patterns of LD [67, 71].

We tested for population bottlenecks using two methods implemented in the program BOTTLENECK v1.2.02 [73]. The first method tested for an excess of heterozygosity relative to observed allelic diversity [74]. We used the two-phase mutation model (TPM), the most appropriate for microsatellites [75], with 70% single-step mutations and 30% of multiple-step mutation. Significance was assessed using Wilcoxon’s signed rank test, as is recommended when fewer than 20 loci are used [73]. The second method tested for a bottleneck-induced mode shift in allele frequency distributions that is usually evident in recently bottlenecked populations [76].

### Hybrid zone analyses

We investigated the mixed ancestry suggested by STRUCTURE analysis in the Achwa and Okole River regions. These sampling sites displayed an average probability of assignment (q-values) of less than 0.8 (See Table 1), which could either be caused by methodological shortcomings (i.e. low genetic distance and inability of the markers to distinguish clear genetic clusters), or by accurate detection of interbreeding of two distinct lineages. Following [77], we tested for accurate detection of interbreeding by comparing observed admixture data against two alternative admixture models; a pure *mechanical mixing* model representing a scenario of strong reproductive barriers and free migration, and a *hybrid swarm* model representing a scenario of free hybridization and admixture using HYBRIDLAB v1.0 [78]. For the *mechanical mixing* model, we simulated individual admixture proportions by randomly drawing alleles from the observed allele frequency distribution of 'pure' samples where the average probability of assignment (q-values) were greater than 0.8 to a single STRUCTURE cluster (Table 1). For the *hybrid swarm* model, we simulated individual admixture proportions from the observed allele frequency distribution of 'admixed' samples where the average probability of assignment (q-values) were less than 0.8 to any single STRUCTURE cluster (Table 1). We chose localities from the geographic extremes of the northwest and northeast units to represent 'pure' samples, and regions with generally uncertain assignment from the Achwa and Okole Rivers to represent 'admixed' samples. Then, we used STRUCTURE to estimate individual probability of assignment (q-value) with all three datasets; the true observed genotypes, the simulated genotypes under a *mechanical mixing* model, and the simulated genotypes under a *hybrid swarm* model. Finally, we used a Wilcoxon signed rank test to assess differences between observed and simulated distributions. We interpret significant differences between simulated *mechanical mixing* and *hybrid swarm* datasets as evidence that uncertain STRUCTURE assignments do not represent a methodological shortcoming. Likewise, we interpret non-significant differences between the observed data and the simulated *hybrid swarm* data as evidence for interbreeding.

### Relatedness and migration

Using microsatellite data, we determined if patterns of observed genetic structure could be attributable to sampling related individuals, by testing for relatedness between individuals using the program ML-Relate [79]. We assigned pairwise relationships within each genetic unit into one of four relationship categories: unrelated (U), half siblings (HS), full siblings (FS) or Parents/offspring (PO).

Detection of first generation migrants and progeny of successful mating of very recent migrants between genetic regions was done using GENECLASS v2.0 [80], and using FLOCK v3.1 [81], a program that provides accurate assignment of individuals to genetic units of origin even in the absence of pure genotypes. In GENECLASS, we used the "detect migrant function", which calculates the likelihood of finding an individual in the locality in which it was sampled (Lh), the greatest likelihood among all sampled localities (L_max_), and their ratio (Lh/max) to identify migrants. To distinguish true from statistical migrants (type I error), we selected the Rannala and Mountain criterion [82], and the Monte Carlo resampling algorithm of [83] (*n = 1000*) to determine the critical value of the test statistics, Lh/Lmax. Individuals were considered immigrants when the probability of being assigned to the reference population was lower than 0.05. In FLOCK, we used a K value of 4, starting partitions chosen by location of origin, ran 500 iterations and used a log-likelihood difference threshold (LLOD) value of 1.

### mtDNA genetic diversity and population structure

For the mtDNA sequence data, all statistical parameters and tests were calculated using the program ARLEQUIN [55]. Genetic diversity within populations was estimated by computing haplotype diversity (H_d_) and nucleotide diversity (N_d_) [84] in DnaSP v5.0 [85]. Relationships among haplotype lineages were inferred by constructing a parsimony network using TCS [86] implemented in the free, open source population genetics software PopART (http://otago.ac.nz). We used nucleotide diversity to estimate genetic differentiation (Φ_ST_) and performed an analysis of molecular variance (AMOVA) in ARLEQUIN. We tested for IBD with the same methods described above for the microsatellites.

Finally, we compared mtDNA haplogroup assignment with the Microsatellite STRUCTURE assignment, and tallied the percent mismatch. Individuals were considered a mismatch if they displayed a high q-value (probability of assignment) score to one microsatellite based cluster but low frequencies of the haplogroup generally found in the same geographic region as the microsatellite based cluster.

## Results

### Microsatellite marker validation

Of the 17 microsatellite markers considered, most were under HW equilibrium in the majority of populations with the exception of pg17, which was dropped from the analyses, as it showed significant departures from HWE at P<0.05. All remaining loci were polymorphic in all populations analyzed except D101, which was monomorphic in one population (OC). The most polymorphic locus was GpB20b (24 alleles), while the least polymorphic was B05 (5 alleles; details in S3 Table). None of the LD tests on pairs of microsatellite loci gave a significant result after the Benjamini-Hochberg correction, confirming neutrality and independence of markers.

### Microsatellite population structure and defining geographic units

**Fig 2:**
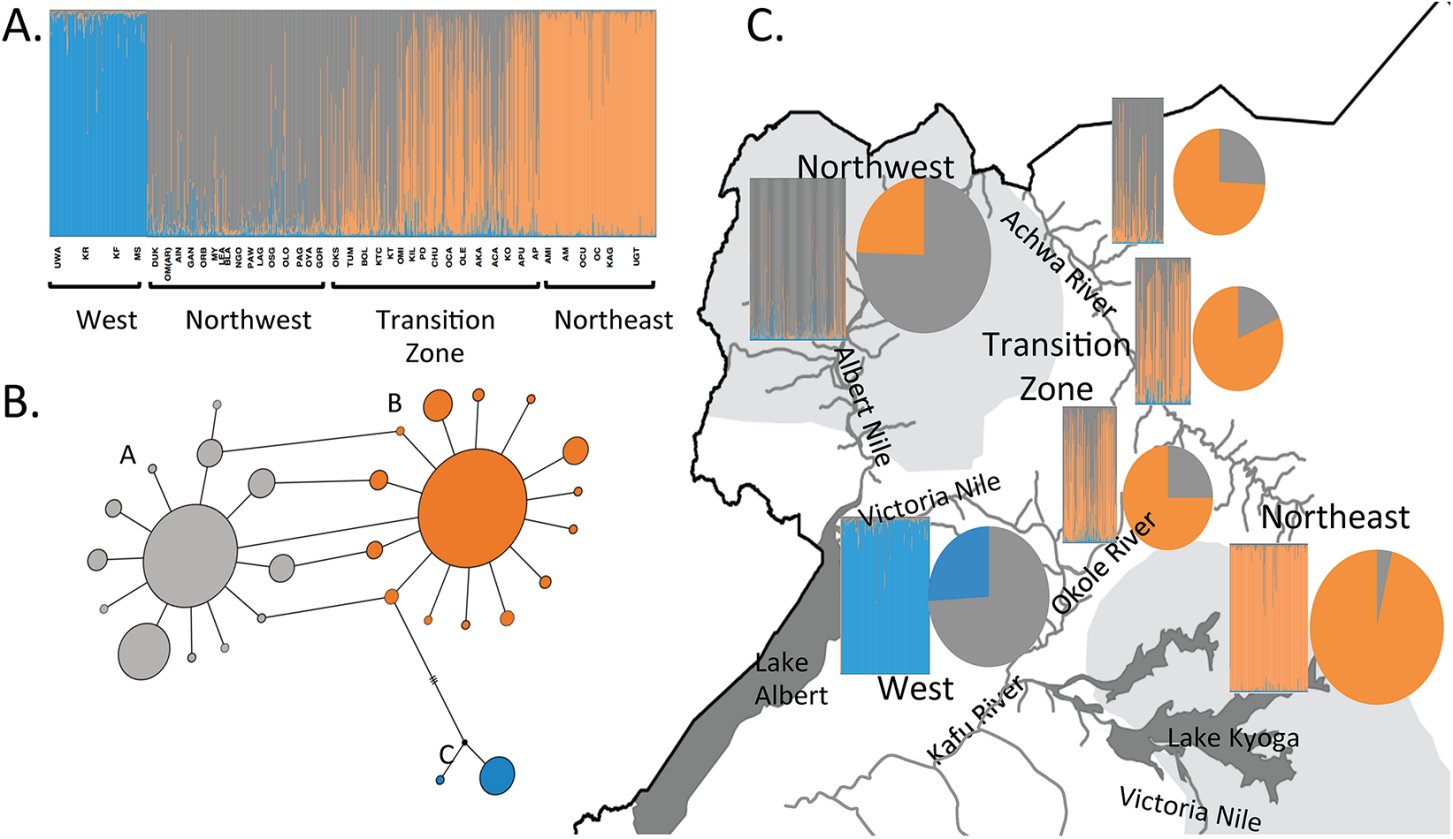
Patterns of genetic differentiation and phylogenetic relationship based on microsatellites and mtDNA markers for *G*. *f*. *fuscipes* in northern Uganda. **(A)** Bayesian clustering plots based on microsatellite data showing genetic membership of 954 flies to three genetic clusters from STRUCTURE v2.3.4 [47]. Y-axis shows the probability of assignment (q-values) for each individual (bars). Colors within each bar represent the probability of assignment to one of the three genetic clusters. Sampling sites and the genetic unit assigned for each individual are reported below the X-axis, using the same abbreviations as in Fig 1 and Table 1. Criteria for assignment of each sampling site to a Genetic Unit are given in the text and in Table 1. **(B)** Haplotype network depicting the mtDNA evolutionary relationships inferred by using the TCS method (Clement *et al* 2002) as implemented in PopART (http://popart.otago.ac.nz). Haplotypes are represented by circles, sized proportional to their frequency in the sample. Dashes have been used to represent mutational steps when haplotypes were separated by more than one step. Haplogroups are identified by different colors; gray (Haplogroup A), orange (Haplogroup B) and blue (Haplogroup C). **(C)** Distribution of the microsatellite clusters (rectangles) and the mtDNA haplogroup frequencies (circles) within the four Genetic Units. Colors are as in panels A and B.

Fig 2A shows the results from STRUCTURE analyses. In this analysis, individuals fell into three genetic clusters with clear geographic variation in probability of assignment (q-value) (Table 1, S4 Table). The DAPC multivariate analysis (S1 Fig) corroborated the results of STRUCTURE.

Based on the results of the STRUCTURE and DAPC analyses and their geographic locations, we grouped sampling sites into four units: West, Northwest, Transition Zone, and Northeast. Sampling sites west of lake Kyoga along the Victoria Nile (i.e. UWA, KR, KF, MS) had average q-values > 0.8 to a single cluster (blue in Fig 2). The samples north of Lake Kyoga (Fig 1) belong to two genetic clusters (gray and orange, Fig 2) and were grouped into three units. The “Northwest” unit comprises samples from the Albert River drainage (e.g. DUK, GAN, and AIN) and the most northerly Achwa River sites (i.e. NGO, LAG and PAW) with average q-values > 0.8 to a single cluster (gray in Fig 2). The “Northeast unit” comprises samples from north of Lake Kyoga (e.g. KAG, AM, OCU and AMI, Table 1) with average q-values > 0.8 to one cluster (orange in Fig 2). Sampling sites between the Northwest and Northeast units in the Achwa and Okole River basins (Fig 1) had a much lower average q-value (0.54) than the other three units, and moving west to east, probability of assignment to one cluster (gray in Fig 2) progressively decreased while it increased for the other cluster (orange in Fig 2). We refer to this region between the Northwest and Northeast units as the "Transition Zone".

Microsatellite based F_ST_ between sampling sites either within or between the Structure-defined clusters ranged from 0 to 0.229 with most comparisons being statistically significant (S5A Table). Table 2 reports average F_ST_ between the four units (Northwest, Transition Zone, Northeast, and West). The West unit is the most genetically distinct from the other three (F_ST_ = 0.162, 0,163, and 0.218 for Northwest, Transition Zone, and Northeast, respectively). Lower but still statistically significant F_ST_ values were estimated between the Northwest and Northeast units (F_ST_ = 0.064) and even lower values between these units and the Transition Zone (F_ST_ = 0.021 and 0.035, respectively). D_EST_ values showed the same trend as F_ST_, except with overall higher estimates (S6 Table). Isolation by distance (IBD) analyses (S7 Table) showed a significant correlation between genetic distance and geographic distance for all sampling sites combined (R^2^ = 0.438, p = 0.0001) and for sampling sites within the Northwest (R^2^ = 0.259, p = 0.00) and Transition Zone (R^2^ = 0.216; p = 0.001). No significant IBD was detected among sampling sites in the Northeast and West units.

**Table 2:** Average estimates of genetic differentiation among the Northwest (NW), Transition Zone (TZ), Northeast (NE) and West (W) units based on microsatellite F_ST_ and mtDNA Φ_ST_ estimates.

Microsatellites				mtDNA			
	Northwest	Transition	Northeast		Northwest	Transition	Northeast
**Transition**	**0.021**			**Transition**	0.041		
**Northeast**	**0.064**	**0.035**		**Northeast**	**0.080**	0.051	
**West**	**0.162**	**0.163**	**0.218**	**West**	0.018	**0.190**	**0.313**

Significant values at p<0.05 are indicated in bold. F_ST_ and Φ_ST_ were calculated in ARLEQUIN *v3*.*5*.

AMOVA results using microsatellites showed that most of the variation was between individuals within sampling sites (89.63%) but differences were statistically significant at all levels of comparison, including between sampling sites and among the four units (Table 3).

**Table 3:** Analysis of molecular variance analysis (AMOVA) based on microsatellite and mtDNA data.

**Microsatellites**					
**Source of variation**	**d.f.**	**Sum of squares**	**Var. components**	**Percentage of variation**	**P-values**
Among units	3	578.062	0.385	7.26	0.000
Among sampling sites within units	38	454.009	0.165	3.11	0.000
Within sampling sites	1866	8855.638	4.746	89.63	0.000
Total	1907	9887.709	5.295		
**mtDNA**					
**Source of variation**	**d.f.**	**Sum of squares**	**Var. components**	**Percentage of variation**	**P-values**
Among units	3	69.464	0.316	32.112	0.000
Among sampling sites within units	37	34.452	0.079	8.048	0.000
Within sampling sites	441	180.280	0.589	59.840	0.000
Total	481	284.196	0.984		

Results of AMOVA within and between the genetic units. Computations were carried out in ARLEQUIN. *v3*.*5*.

### Microsatellite genetic diversity

Overall, all sites showed moderate to high levels of genetic variability (S1 Table). H_O_ ranged from 0.461 in LAG to 0.690 in OM and H_E_ ranged from 0.537 in KAG to 0.678. For most of the sites, H_O_ and H_E_ microsatellite diversities were similar, indicating random mating within sites. Averaged over all samples and loci, the inbreeding coefficient (F_IS_) were generally low with an overall grand mean of 0.048±0.008, and with significant heterozygote excess in 7 out of 42 populations (S1 Table). Allelic richness ranged from a high of 7.785 in KR to a low of 4.188 in AMI, with an overall mean of 5.186 (S1 Table). Generally, microsatellite diversity was highest in flies sampled in the Northwest and the Transition Zone sites (Table 1; S1 Table) and lowest in flies from the Northeast (e.g. in sites KAG, AM, OCU and AMI). The trend of decline in diversity from the Northwest to the Northeast is apparent and significant when allelic richness values were linear-regressed over longitude (R^2^ = 0.121; p = 0.032; S2 Fig). Flies in the West unit had microsatellite diversity values similar to or on par with the Northwest unit.

### Effective population size (Ne) and bottleneck analysis

S8 Table shows the results of the Ne estimates based on microsatellite data using the LD and the temporal methods. Estimates using the heterozygote excess method were infinite for all sites tested. Using the one-sample LD method, Ne estimates ranged widely from 101.6 (36.4-infinite 95% confidence interval [CI]) in OC to 1685.7 (234.2-infinite CI) in UGT and were all bound by a CI that included infinity (S8 Table). Ne estimates using the two-sample temporal method ranged from 103 (73–138 CI) in KTC to 962 (669–1309 CI) in OCU (S8 Table). Where a comparison between the two methods was possible, estimates were largely congruent except for one site (OCU), where Ne using the temporal method was 962 (669–1309 CI), and using the LD method was 112 (47.7-infinite CI; S8 Table). Results based on the TPM model indicate a genetic bottleneck in 5 sampling sites (NGO from the Northwest, OCA from the Transition zone, AMI and OC from the Northeast, and MS from the West; S8 Table). Results based on allele frequency distributions showed a genetic bottleneck in only one sample (AMI from the Northeast; S8 Table).

### Hybrid zone analyses

**Fig 3:**
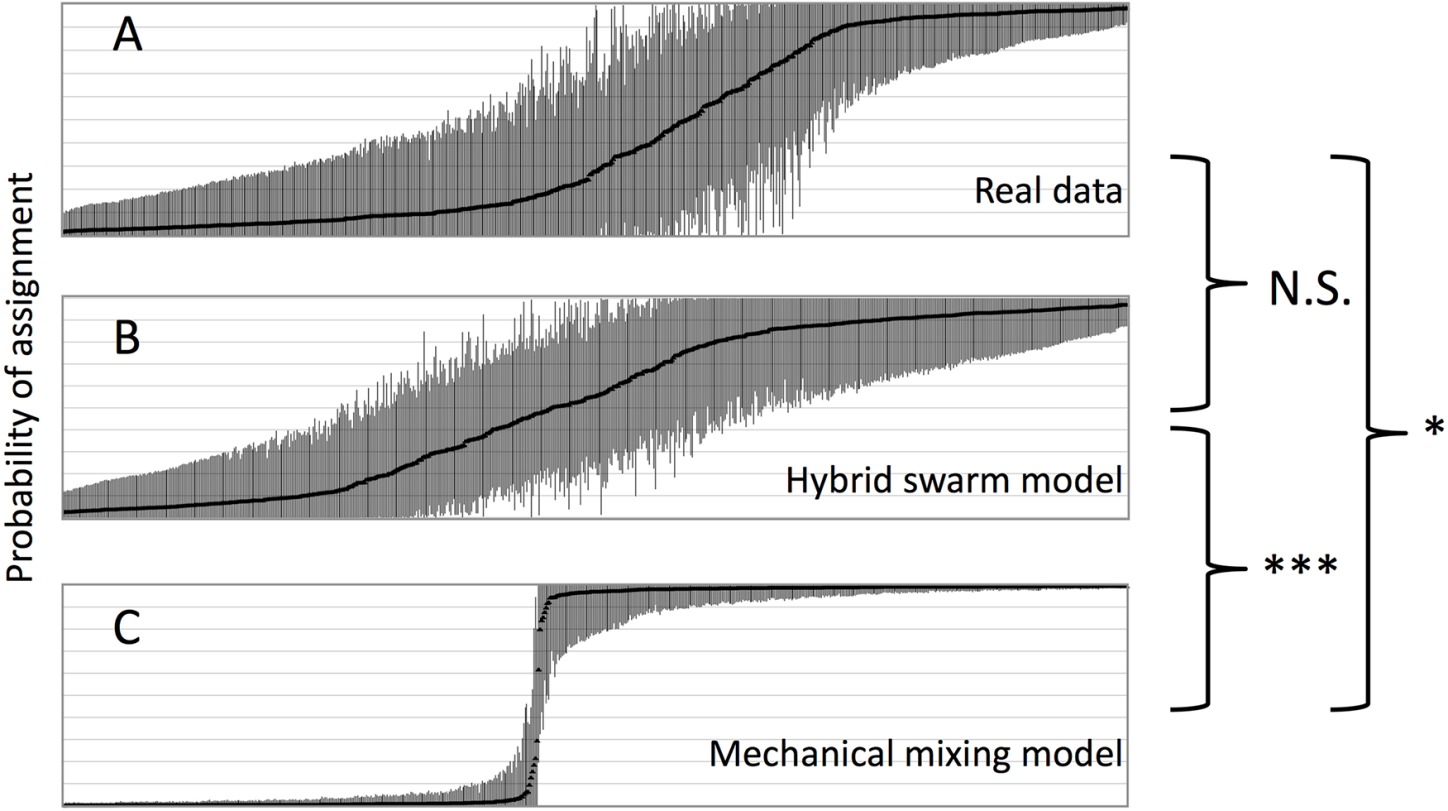
Hybrid zone analysis. Distribution of probability of assignment (q-values) and 95% posterior probability intervals among individuals estimated using STRUCTURE for; **(A)** the true observed genotypes, **(B)** the simulated genotypes under a *mechanical mixing* model, and **(C)** the simulated genotypes under a *hybrid swarm* model. Highly significant difference (p-value <0.0001) are marked by ***, significant difference (p-value <0.05) are marked by *, and non-significance is indicated as "NS".

Fig 3 shows the results of the HYBRIDLAB analyses. The distribution of STRUCTURE assignments from the simulated *hybrid swarm* and *mechanical mixing* datasets are clearly distinct (Fig 3) with a Wilcoxon two-tailed p-value of 0.002 (S9 Table). This indicates power to detect interbreeding in the transition zone and thus evidence of hybridization. Comparisons of these models with the observed data (S9 Table) indicate that the observed data (Fig 3A) matches most closely with the *hybrid swarm* model (Fig 3B) than the *mechanical mixing* (Fig 3C) from which it is statistically distinct.

### Relatedness and migration

Relatedness analyses showed that the majority (>86%) of the individuals in all units are unrelated (Table 4). The percentage of individuals that were full siblings was very low, ranging between 0.33% and 0.91% for all units. An even lower number of individuals had parent-offspring relationships ranging from 0% in the Transition Zone to 1.04% in the Northeast.

**Table 4:** Relatedness category for *G*. *f*. *fuscipes* samples within the four units and combined.

	Northwest	Transition	Northeast	West	Overall
**Unrelated**	87.93	90.33	82.90	86.04	86.93
**Half siblings**	11.51	9.33	15.15	13.09	12.35
**Full siblings**	0.49	0.33	0.91	0.67	0.61
**Parent-Offspring**	0.07	0.00	1.04	0.20	0.11

Percent of pairwise comparisons of individuals that fell into each relatedness category as calculated in ML-Relate [79]. Comparisons were made among the 3 units Northwest, Northeast, West) and the Transition zone; and among all samples combined (Overall).

**Fig 4:**
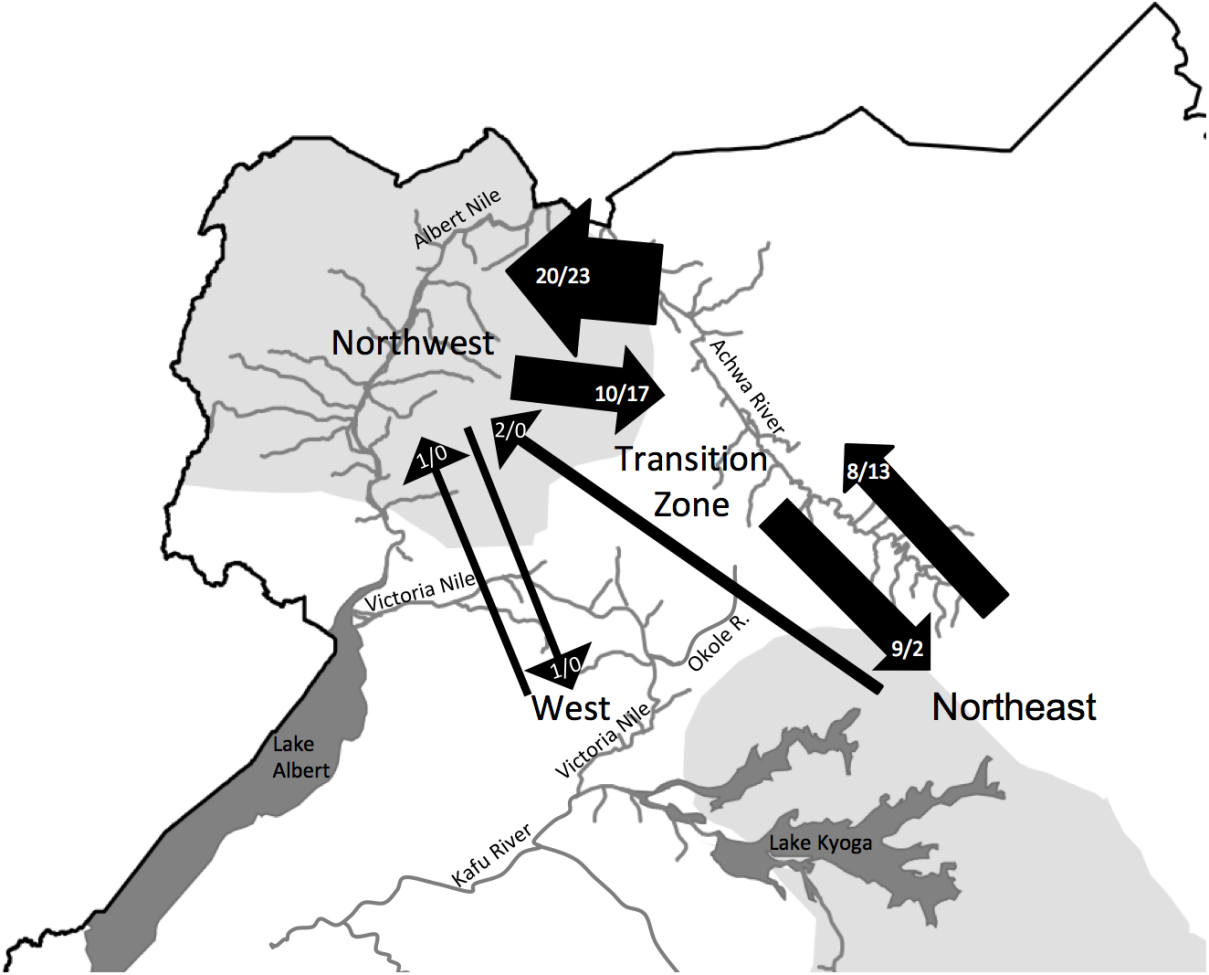
Migration patterns. Arrows summarize the direction of movement of first generation migrants and progeny of successful mating of very recent migrants obtained using the program GENECLASS [80] and FLOCK [81] respectively, separated by a slash. Their thickness reflects the relative amount of migrants with actual estimates reported above each arrow.

Microsatellite-based migrant detection using GENECLASS and FLOCK showed a higher number of migrants between the Northwest, the Transition Zone, and the Northeast than between these areas and the West (Fig 4). GENECLASS indicated slight asymmetry in migration into the Northwest. There are 20 migrants from the Transition Zone into the Northwest and 10 migrants in the reverse direction, with both sexes almost equally represented (10 and 2 male migrants *vs*. 10 and 6 female migrants; S10 Table). We also detected two first generation female migrants from the Northeast to the Northwest. In contrast, migration between the Transition Zone and the Northeast is symmetrical with 8 migrants from the Northeast into the Transition Zone and 9 migrants in the opposite direction. Both sexes are moving in both directions, although the low sample sizes (2 and 3 male migrants *vs*. 5 and 1 female migrants; S10 Table) precludes any strong conclusion. We also detect two migrants between the Northwest and West, one in each direction. FLOCK analysis provided similar migration rates between regions (Fig 4), but showed less asymmetry from the Transition Zone into the Northwest (23 from the Transition Zone into the Northwest, and 17 in the opposite direction), and more asymmetry from the Northeast into the Transition Zone (13 from Northeast into the Transition Zone, two in the opposite direction; S10 Table). FLOCK showed no migration between the West and any other region (S10 Table).

### mtDNA genetic diversity and population structure

The mtDNA dataset consisted of 481 sequences (490 bp long), which included 289 sequences from individuals sampled for this study (a subset of the ones screened for microsatellite loci variation, Table 1) plus 192 sequences from individuals from previous ones [25][31], Table 1). Sequences could be grouped into 30 haplotypes (S11 Table), displayed as a TCS network (Fig 2B). There are three major haplogroups (groups of related haplotypes); Haplogroup A, Haplogroup B, and Haplogroup C (Fig 2B). Table 1 reports haplogroup frequencies for each site and for the four units. Haplogroup A occurs in all studied regions, but is most frequent in the Northwest and West units (75.1% and 74.4%, respectively). It occurs less commonly in the Transition Zone (24.0%) and only rarely in the Northeast (4.8%). Haplogroup B occurs most commonly in the Northeast unit (95.2%) and it is less common going from Northeast unit to the Transition Zone (76.0%) and to the Northwest unit (24.9%). Haplogroup B does not occur in the West unit and Haplogroup C occurs only in this unit (25.6%, Table 1, Fig 2C).

The number of haplotypes at each sampling site ranged from 1 to 6 (S1 Table). Haplotype 1 is the most frequent (186 individuals) and occurs in all units except the West, and falls into Haplogroup B (S11 Table). Haplotype 2 from Haplogroup A is the second most common (140 individuals) and it is found in all units (S11 Table). The third and fourth most common haplotypes fall in Haplogroup A and C, and only occur in the West (41 individuals and 19 individuals, respectively). Thirteen haplotypes were singletons (observed once in the sample) and fall into a mix of haplogroups, eight of which were from the Northwest, four from the Transition Zone and one from the West (S11 Table). Nucleotide diversity averaged 0.002 and ranged from 0 (OSG and KF) to 0.008 (UWA; S1 Table). Likewise, average haplotype diversity was 0.757, and ranged from 0 (KF and OSG) to 0.836 (UWA; S1 Table). There was no apparent difference in haplotype diversity from Northwest to Northeast units.

S6B Table shows estimates of genetic differentiation (Φ_ST_) between sampling sites. Φ_ST_ ranged from 0 to 1; with some sampling sites showing no evidence of differentiation (e.g. PD in the Transition Zone *vs* AMI in the Northeast), while reached 1 for pairs that did not share haplotypes at all (e.g. OSG in the Northwest *vs* KF in the West). S7 Table shows the results of the IBD analyses using mtDNA-based Φ_ST._ Like in the microsatellite-based test, the correlation between genetic distance and geographic distance was significant for all sampling sites combined (R^2^ = 0.490, p = 0.001) and for samples within the Northwest unit (R^2^ = 0.425, p = 0.001), but non-significant for the Northeast and West units. Unlike in the microsatellite-based IBD tests, the correlation between geographic and genetic distance in the Transition Zone was non-significant (R^2^ = 0.002; p = 0.374).

AMOVA results based on mtDNA agree with the Microsatellite (Table 3), with most of the variation between individuals within sites (59.84%) and significant values at all levels of comparison, including between the four units (Northwest, Transition Zone, Northeast, and West; Table 3).

To evaluate the possible role of differential introgression of nuclear *vs* mitochondrial markers we assessed levels of mismatches by comparing individual assignments for each marker type (S4 Table), and calculated percent individuals with discordant nuclear *vs* mitochondrial assignment (Table 1). This analysis could only be done for the three northern units because the common microsatellite based cluster (blue in Fig 2) in the West was not clearly associated with a single mitochondrial haplogroup, as both Haplogroup A and Haplogroup C occur there. On the contrary, the Northeast and Northwest were clearly associated each with a single haplogroup, so we scored any individual from the north with a microsatellite-based q-value greater than 0.9 as a “match” if both nuclear and mitochondrial assignments were associated with the same region (grey/grey or orange/orange in Fig 2), or a “mismatch” if assignments were associated with different regions (grey/orange or orange/grey in Fig 2). The highest percentage of mismatches were found in the Northwest unit (22.8%), followed by the Transition Zone (20.03%), and then the Northeast unit (4.0%) (Table 1).

## Discussion

We evaluated the fine scale genetic structure of *G*. *f*. *fuscipes* populations north of Lake Kyoga in Uganda, a region that is of special interest due to the impending risk of merger of the two forms of HAT disease that *G*. *f*. *fuscipes* transmits in Uganda. Our sampling scheme targeted the fine spatial scale resolution of genetic structure so as to provide the most accurate information available on genetic connectivity and population dynamics in the region spanning the two HAT disease foci. This kind of information is necessary to inform vector control program design [87–91]. Findings indicate two strong genetic breaks in northern Uganda and determine that hybridization is occurring freely across the contact zone between the Northwest and Northeast. We explored underlying mechanisms of population dynamics in northern Uganda and found that large influence has been imposed by (i) sustained connectivity of the Northwest with the rest of the *G*. *f*. *fuscipes* species range, (ii) past geologic events associated with the opening of the great rift valley during the last ~35 ka, and (iii) vector control programs that have caused population bottlenecks but have not always sustainably controlled tsetse populations. We also identified a general pattern of isolation by distance and moderate migration within interconnected regions. Findings suggest that population rebounds may have occurred from very close by populations soon after vector control efforts, and that interbreeding across a hybrid zone that spans the two disease foci could promote recolonization from different genetic units across further distances. These results support the need for long-term monitoring and a design that mitigates recolonization from neighboring regions, especially within the hybrid zone that spans the two disease foci.

### Patterns of genetic diversity and population dynamics

Genetic diversity at both microsatellite and mtDNA markers (Table 1) were generally low compared to many Diptera and Coleoptera species, which is consistent with reproductive limits imposed by the tsetse's viviparous life history [92]. The mtDNA haplotype network (Fig 2B) was congruent with the network published by [24] with more haplotypes because of the higher spatial resolution of this study. Levels of diversity in both markers (Table 1) were similar to previous estimates for sampling sites north of Lake Kyoga [25][31], but higher than estimates of southern Uganda populations [27]. We found a subtle decline in genetic diversity from west to east (S1 Fig) in northern Uganda similar to the pattern previously observed in central and southern Uganda [25]. [25] suggested that this gradient reflected sequential founder events originating from the main tsetse belt in the Northwest and moving eastward. Conversely though, our results for northern Uganda are not consistent with a single genetic origin from the main tsetse belt because we found two distinct genetic backgrounds (Fig 2A) and two distinct mtDNA haplogroups (Fig 2B). This apparent inconsistence between past and current results could be due to the inability of previous studies to pick up the spatial differentiation and admixture patterns that we detected because of their much sparser sampling than in this study. Rather than sequential founder events pushing for a northwest to northeast range expansion, our results suggest that sustained connectivity to the greater *G*. *f*. *fuscipes* distribution and recent human induced population processes, such as vector control and habitat destruction, may account for the higher genetic diversity in the Northwest *vs* the Northeast.

The *G*. *f*. *fuscipes* range extends continuously westward as far as Cameroon and Gabon (Fig 1; [93][94]) and has been sustained since the last glacial maximum ~15–20 ka [95][2][96], with the Uganda sites being at the extreme northeast of *G*. *f*. *fuscipes’* contiguous distribution. The size of this range and its temporal stability suggest that populations from the main part of its distribution are likely to be interconnected and old enough to harbor the high levels of genetic diversity characteristic of large and stable populations. This may have facilitated intermittent gene flow and can be a factor in explaining the higher genetic diversity in the Northwest than in Northeast of Uganda. In contrast, populations to the east and south of Lake Kyoga are bordered by unsuitable habitat to the east [93], and have experienced recent arid periods during the last glacial maximum ~15–20 ka, and again during the latest Pleistocene ~14 ka, when the lakes in Uganda completely desiccated multiple times [97][95]. These climate events could have led to contractions and expansions of populations, accentuating the effects of genetic drift and creating isolated populations with low genetic diversity such as that found in UGT, AMI, AM, OC, KAG, and OCU (Table 1; S2 Fig). Despite high genetic diversity in some localities in the West such as UWA (Table 1; S2 Fig), which conforms with the general pattern of high to low diversity from west to east (S2 Fig), connectivity with the rest of the *G*. *f*. *fuscipes* distribution in the West is limited by Lake Albert and the less suitable habitat in the bordering Blue Mountains. Thus, we suggest that the high allelic richness and haplotype diversity in the West was created by contact between the distinct genetic lineages at the Victoria Nile with a small amount of asymmetrical introgression (see below) rather than through connectivity with the central part of the species range.

As expected, the gradient from higher to lower effective population size estimates (Ne) from the northwest to the southeast parallels the results on genetic diversity, and is likely caused by similar evolutionary forces, as Ne calculations are based on diversity estimates. Our interpretation of Ne was somewhat limited because we were only able to draw inference from the two-sample temporal method. As expected, the one-sample LD based Ne estimates yielded high confidence intervals that overlapped with infinity (S8 Table). Improved Ne estimates based on a larger number of nuclear markers will be an important focus of future research using Single Nucleotide Polymorphisms (SNPs). Despite uncertainty in Ne estimates from the LD method [67], the temporal method [64] provided estimates that ranged from 100 to 1000, had low confidence intervals (S8 Table), and showed higher estimates in the Northwest. This result is in line with the distinct life-history traits of tsetse flies such as lower population sizes, reproductive outputs, and longer generation times than other insects. Ne and genetic diversity results that we report for the Northwest were similar to what has been reported in *G*. *f*. *fuscipes* sampled from northern Uganda [31][25] and in *G*. *palpalis*, another riverine species [1]. However, estimates were higher than reported in populations from southern Uganda [27]. This suggests that Northwest populations are influenced by either high connectivity with the rest of the *G*. *f*. *fuscipes* range, or by lower levels of vector control in the Northwest as compared to regions impacted by the SOS campaign in the Northeast.

Detection of recent bottlenecks (S8 Table) provides further evidence that Ne has been influenced by vector control campaigns. The bottleneck analysis we used can detect extreme reductions in population sizes that occurred more recently than 2–4 Ne generations [67][98], which corresponds to 25–500 years in *G*. *f*. *fuscipes*, depending on the exact Ne and generation time of the population in question [99][25][31][27]. Signals of bottlenecks from these short time scales can be due to natural or human induced changes in population size. Both of these causes may be at play given *G*. *f*. *fuscipes’* patchy distribution, unique life history traits, and history as the target of intense, even if somewhat irregular, vector control campaigns. Bottlenecks in OC and AMI can be attributed to the SOS campaign of 2009 [100][41]. Similar tsetse control projects, like the Farming in Tsetse Controlled Areas (FITCA) in southeastern Uganda in places not included in this study like Okame, Otuboi, and Bunghazi, resulted in detection of bottlenecks in these areas in previous studies [26][25]. On the other hand, we found no evidence of bottlenecks in the 2014 samples most proximal to the location of the pilot vector control program conducted by [39] in the district of Arua (DUK, AIN and GAN). This may have been because the location of sampling was too spatially distant (minimum of ~20 km) to influence the population sampled, or because the time of sampling was too temporally near (~6 months) for a genetic signal to propagate. Survey data indicates that some control efforts resulted in long-term reduction in tsetse census [26,31], while other control efforts such as the SOS campaign in the Northeast resulted in only short-term population size reductions. Population rebound is evidenced by the similar number of flies caught per trap at sites in the SOS region and at sites where no control activities have been carried out. For example, during sampling in 2014, traps set at two sites in the SOS region (OCU and AMI) caught an average of 67 and 14 flies per trap, which are numbers that are similar to or higher than the average catch of 18 flies per trap. This underscores the importance of long-term control and monitoring campaigns following tsetse control. A population bottleneck in GOR (S8 Table) remains unexplained by known vector control campaigns, which may indicate that some bottlenecks are induced by natural evolutionary processes such as weather events or changes in ecological interactions. Thus, there is evidence that the joint influence of natural processes such as the greater connectivity to the rest of the *G*. *f*. *fuscipes* distribution in the Northwest, the dramatic climate change including arid periods in central and southern Uganda in the last ~35 ka years, and recent vector control programs have determined the west to east gradient in *G*. *f*. *fuscipes* genetic diversity and population dynamics in northern Uganda.

### Patterns of genetic differentiation

Clustering (Fig 2A) and multivariate (S1 Fig) analyses detected three distinct genetic clusters each composed of multiple sampling sites and broadly corresponding to the Northwest, Northeast, and West units (Fig 2C). mtDNA haplotypes also clustered into three haplogroups (Fig 2B), which approximately correspond to these same three regions (Fig 2C). Two of these genetic lineages have been described by previous research as Northern and Western clusters [24][25][31], and we find evidence of a previously undescribed divergence in the Northern cluster, which is now partitioned into Northeast, Transition Zone, and Northwest units.

Our data confirm deep genetic divergence between the *G*. *f*. *fuscipes* nuclear lineages found at the Victoria Nile, which harbor a mix of mtDNA haplotypes of both northern and southern associated lineages (Fig 2). STRUCTURE clustering showed close geographic proximity of distinct clusters at the confluence of the Okole River and the Victoria Nile (Fig 2). This stark genetic break in the nuclear genetic make-up may be due in part to insufficient sampling between UWA in the West and AKA and OLE in the North for accurate description of the shape and geographic span of the genetic divergence between these regions. Future sampling efforts should encompass detailed sampling in this region to determine if there is indeed another transition zone between the West and units identified in this study (i.e. Northwest, Transition Zone, and Northeast), and between the West and previously described units [24][25][31]. Divergence between the North and West is thought to have originated during past allopatry more than 100,000 years ago [25]. Subsequent changes in the river systems associated with the opening of the great rift valley 13,000–35,000 years ago [95] created the modern outflow from Lake Victoria into Lake Kyoga and the reversal of the Kafu river to meet the Victoria Nile before flowing into Lake Albert [97]. These changes may have shifted the range of the Western *G*. *f*. *fuscipes* populations into contact with the Northern units at the Victoria Nile.

We find mixed mtDNA ancestry in the West, which [24] described and suggested indicates recent rare female dispersal from the north and chance amplification of northern haplotypes by drift. Another possible explanation is the preferential introgression of organelle DNA from the resident population into the colonizing genetic background when two divergent lineages come into secondary contact during range expansion [101]. This scenario is supported by changes in the river systems and multiple drying cycles of the lakebeds [85] that would have promoted repeated retraction and expansion of *G*. *f*. *fuscipes* in central Uganda. If northern lineages had recolonized central Uganda before a northward shift of the southwestern lineage, the result would be a large number of northern mtDNA haplotypes in a Western nuclear genetic background. There are also possible ecological interactions at play because this region is unique in the co-distribution of other tsetse species, *G*. *morsitans submorsitans* and *G*. *pallidipes* [2] especially in the large protected area of the Murchison Falls National Park (Fig 1). Thus, evidence supports that strong evolutionary and ecological forces maintain genetic distinctiveness between the West and the other genetic units, but the details remain unclear and an important focus for future work. More fine scale sampling of all tsetse species across the North/West genetic break as well as experiments that test mating compatibility would help shed light on the mechanism(s) that maintain genetic discontinuity.

Both microsatellite based F_ST_ and mtDNA based ɸ_ST_ showed significant differentiation despite our fine scale sampling effort, which aligns with previous studies that have found significant differentiation across small geographic scales of as little as 1–5 km^2^ in Uganda [102][27][25]. Tsetse flies are known to be sensitive to environmental conditions and exist in discrete patches [2]. We suggest that low connectivity between adjacent habitat patches coupled with small Ne has allowed genetic drift to create significant differentiation at small spatial scales in *G*. *f*. *fuscipes* in northern Uganda. High signals of isolation by distance we detected in both microsatellites and mtDNA (S7 Table) further support the idea that population structure is maintained by the dual action of migration and genetic drift.

### Levels of genetic admixture

The genetic break between the Northwest and Northeast forms a broad region of mixed microsatellite and mtDNA assignment along the Achwa and Okole rivers, in what we call the Transition Zone. The genetic break between the Northwest and Northeast and the one between the broad northern and southern clusters described by [25] are both characterized by what we think are secondary contact with admixed individuals and introgression of mtDNA haplotypes. However, the width of the transition zone, the level of differentiation, and the patterns and levels of admixture, is different across these two contact zones, with a broader, less differentiated, and more gradual pattern of admixture in the Transition Zone than in the North/South contact. The Transition Zone extends more than 200 km (Fig 2), while the secondary contact zone between the North and South clusters extends less than 75 km [25][31]. This difference in width may have been facilitated by colonization patterns and the distinct geographical break imposed by the swampy upper reaches (southern extent) of Lake Kyoga at the contact zone between the North and South, while less conspicuous physical breaks only partially limit movement of flies to and from the Transition Zone (Fig 1). The Transition Zone is characterized by uninterrupted suitable habitat along the entire length of the Achwa River, with only short distances of less than 15 km between the Achwa and Okole Rivers and neighboring drainage basins of Lake Kyoga and the Albert Nile (Fig 1).

The levels of differentiation are also different between these two contact zones. In the Transition Zone, microsatellite-based F_ST_ estimates are lower (average F_ST_ = 0.064, Table 2) than the comparable values for the North and South clusters (average F_ST_ = 0.236; [25]). This pattern was even more extreme in mtDNA Φ_ST_ estimates, with an average Φ_ST_ of 0.080 between the Northwest and Northeast (Table 2) and 0.535 between the North and South [25][103][31].

Similarly, the patterns of admixture are distinct between the two contact zones. In the North/South contact zone, there is a dramatic increase in mismatched individuals that assign with high frequency (>90%) to one nuclear based genetic cluster but with mtDNA haplotypes found in another [31] at the zone of contact, with 16.98% in the contact zone *vs* 0–2% on either side. The pattern of mismatch in the North/South contact zone suggest differential introgression of mtDNA and nuclear loci, which could be due to *Wolbachia* infections [25][104][103][31], given its maternal inheritance and ability to induce cytoplasmic incompatibility in *G*. *morsitans* [105] and other insects [106]. In contrast, in northern Uganda, the Transition Zone does not display an increase in mismatches, with 19.9% in the contact zone *vs* 26.2% to the north, which leaves no evidence of differential levels of introgressions of the two markers (Table 1) or asymmetrical introgression. The match of observed data with the *hybrid swarm model* in the HYBRIDLAB analysis (Fig 3) provides further evidence of relatively free and symmetrical interbreeding in the Transition Zone over multiple generations.

Taken together, our results suggest that for the northern secondary contact area, isolation by distance and genetic drift are the two most likely processes that have shaped the distribution of the nuclear and mtDNA polymorphisms, rather than *Wolbachia* infections. Nonetheless, symmetrical interbreeding in the Transition Zone of this study remains tentative without the ability to classify hybrid classes because of wide and overlapping 95% confidence intervals around expected q-values (Fig 3A). Further genetic characterization of the northern hybrid zone as well as characterization of the circulating *Wolbachia* strain(s) in the North would improve understanding of the forces shaping the genetic cline that lies between the disease belts of the two forms of HAT in northern Uganda.

### Migration patterns

The methods we used to detect migrants reflects both first generation migrants and progeny of successful mating of very recent migrants rather than dispersal, and thus allowed us to assess recent gene flow across the full geographic range of our study [82][81]. We detect comparatively high migration rates among the northern clusters and low migration between these units and the West. High gene flow between the three northern units supports the assertion by [91] and others that waterways, in this case the Achwa River, maintain connectivity in tsetse populations. The vast majority of the migrants were a result of short-range dispersal from geographically proximate sampling sites connected by rivers. GENECLASS detected only two long-range migrants from the Northwest into the Northeast, which would not be expected with available ecological and physiological data that indicate tsetse cannot disperse over long distances [107]. Thus, it is likely these long-range migrants are offspring of assortative mating between first generation migrants found in geographically intermediate locations rather than first generation migrants.

The overall direction of migration we detected was slightly asymmetrical towards the Northwest from the Northeast. However, we found no evidence of sex-bias (S10 Table). These findings agree with previous studies which detected similar movement rates for the two sexes for *G*. *f*. *fuscipes* from the southeast into the northeast [25][31]. [25] suggested that movement along riverine habitats might be linked to passive dispersal of pupae via seasonally flooded river systems. Transportation of adults and pupae downstream may also be aided by large floating islands with dry substrate that form in backwaters and eddies and move northwards for sometimes hundreds of km along the major rivers in the region such as the Nile and its tributaries, and potentially, the Achwa river [108] [109]. Nonetheless, this hypothesis remains to be tested, and alternatives include the movement of flies with livestock [3][110], shifting distribution of suitable habitat with human activities, and ongoing migration along corridors of suitable habitat that connect the north and south of Uganda.

### Implications for vector control and future directions

The observations from this study have important implications on the epidemiology of the two HAT diseases, as well as on future vector control and monitoring efforts in this region. A dense sampling scheme across a relatively small geographic area allowed an unprecedented spatial resolution of genetic structure in this region. Our results point to the presence of four genetic units, three of which have high levels of gene flow among them. The genetic distinctiveness of the West from the other three units suggests that this unit could be treated as a separate entity from the Northern ones. However, when planning control and monitoring strategies, it is opportune to look at the patterns and levels of genetic discontinuities between West vs. South and West vs. North genetic backgrounds in more detail to more precisely define the boundaries of each genetic unit at a country-wide scale. Given the results of this work, for sampling sites North of Lake Kyoga, control efforts undertaken at the unit level are unlikely to produce long-lasting results due to re-invasion from adjacent units, unless physical barriers are incorporated to avoid re-invasion from adjacent units. The best strategy would be a “rolling-carpet” initiative where control is initiated from the Northeast through the Transition Zone into the Northwest followed by intense monitoring and additional control to manage fly migration from previously cleared sites due to population recrudescence after control.

Our results suggest that ecological and geographic features, especially the river systems in northern Uganda, play a major role in keeping *G*. *f*. *fuscipes* populations connected–a fact that should be taken advantage of when designing control. The genetic connectivity we found along waterways provides further support for a vector control strategy that incorporates targets along waterways and barriers to recolonization from adjacent stretches of riverbanks. This idea is also supported by a recent study that comprehensively evaluated a “tiny targets” vector control strategy along riverine savannah and found that a target density of 20 per linear km can achieve >90% tsetse control [39].

Our data also suggest that there is current movement of flies from the Northeast and Northwest into the Transition Zone but with a slight asymmetry towards the Northwest. Given that previous studies also demonstrated northward migration from the east [25][31], it is possible that tsetse, besides livestock movement, is contributing to the northwards expansion of the *T*. *b*. *rhodesiense* sleeping sickness.

Of major relevance for disease control is the finding of high levels of genetic intermixing and gene flow in the Transition Zone, which implies that a fusion of the two diseases (*T*. *b*. *gambiense* and *T*. *b*. *rhodesiense*) is unlikely to be prevented by an incompatibility between vector populations in the region of contact. Given the extent of connectivity in the three northern genetic units and the apparent genetic stability of *G*. *f*. *fuscipes* populations in the region [37], ongoing monitoring following control would be paramount if interventions are to be sustainable. Monitoring programs should involve a combination of both ecological and genetic surveys to check on changes in population density and re-emergence either from residual pockets of tsetse or dispersal from proximal locations. For example, our results from the Northeast highlight the risk of population rebound following control. In this region, we found strong evidence of genetic bottlenecks indicating initial success of the SOS campaign in reducing tsetse density. However, our 2014–2015 surveys in the same sampling sites returned some of the highest tsetse trap densities. It appears, therefore, that when control activities were relaxed at the end of the SOS campaign, tsetse populations recovered to high densities. Focused monitoring could provide early detection of such population rebound and allow for identification of the source and proper mitigation of the recolonizing tsetse.

## Supporting information

S1 Fig Discriminant Analysis of Principal Components (DAPC) based on genetic diversity at 16 microsatellite loci in 42 populations and obtained using the *adegenet* package [52] in R [53]. Two linear discriminants (LD1 and LD2) were used, following selection of principal components using a-score optimization, to plot *G*. *f*. *fuscipes* genotypes. Color codes are the same as in Fig 3. Letter codes represent sampling locations. Dots represent individual genotypes and the groups belonging to a sampling site as ellipses. Upper and bottom left insets show eigen values of principle components in relative magnitude. Black bars of eigen values show the proportion of principal components retained.
(PDF)

S2 Fig Linear regression of allelic richness (microsatellite loci) and haplotype diversity (mtDNA) over longitude produced using JMP V11.0 (SAS Institute Inc., Cary, NC, USA, 1989–2007). Triangles, diamonds, and squares identify sampling sites within the Northwest, Transition Zone, Northeast genetic units, respectively.
(PDF)

S1 Table Sample information and molecular diversity indices. Sample geographic locations, sample sizes, and genetic diversity statistics for 16 microsatellite loci and for a 490bp mtDNA COI-COII gene fragment in 42 populations of *G*. *f*. *fuscipes*. ** Indicates samples collected prior to 2014, N = number of individuals analyzed, AR = Allelic richness, Ho = Observed heterozygosity, He-Expected heterozygosity, Fis = inbreeding coefficient.
(XLSX)

S2 Table Microsatellite loci information. The table reports loci names followed by the forward (F) and reverse (R) primers names and sequences. The last column reports its source. M13 tails are marked with an asterisk (*).
(DOCX)

S3 Table Total number of microsatellite alleles by locus. (DOCX)

S4 Table Table shows the probability of assignment (q-values) of individuals to each of the 3 genetic units, individual mtDNA haplotype, home region of individual, and if it’s a migrant the origin of migration, as well as comparison between microsatellite and mtDNA genetic assignment. (XLSX)

S5 Table Pairwise F_ST_ and ɸ_ST_ comparisons for microsatellites (A) and mtDNA (B) respectively. F_ST_ values are reported in the lower diagonal. Since most values are significant, we highlight those that are **non-significant** in **bold**. All computations were done in ARLEQUIN. Significance was calculated based on a P<0.05.
(XLSX)

S6 Table Pairwise D_EST_ for 42 populations averaged over 16 loci. The first two columns show the sampling site pairs, while the third and fourth columns report their mean D_EST_ values and the Benjamini-Hochberg corrected significance p-values, respectively. Estimates were made in the R package DEMEtics [60].
(XLSX)

S7 Table Results of tests for isolation by distance where geographic distance between sampling sites (km) were linear-regressed over nuclear microsatellites based genetic distance (F_ST_ /(1-F_ST_)) and mtDNA sequence based genetic distance (Φ_ST_/(1-Φ_ST_)). Results for the Northwest, Transition Zone, Northeast, West units, and all samples combined (Overall) are shown separately. Genetic group, root mean square values (R^2^), p-value for the Mantel test (p), and slope and intercept of the linear regressions are shown. Significant correlations are indicated in bold (p<0.05).
(DOCX)

S8 Table Effective population size and bottleneck tests. Estimates of effective population size (Ne) were computed for each of the 42 sampling sites across the geographic regions in northern Uganda using three methods: LD, modified temporal method of Waples [66] based on [67] and heterozygote excess method. Estimates are provided together with their 95% CI. Bottleneck tests were carried out using the two methods implemented in the program BOTTLENECK [73]. Significance of tests for population bottlenecks assumed TPM model and it is displayed as a p-value based on 1-tailed Wilcoxon's test (P<0.05).
(XLSX)

S9 Table Wilcoxon signed rank test results comparing STRUCTURE results from the real data to the *hybrid swarm model* and the *mechanical mixing model*. (XLSX)

S10 Table Table showing the total number of migrants based on GENECLASS and FLOCK. General information is displayed (population, closest village, drainage basin). Total, female, and male migrants are shown as counts from each unit based on both analyses.
(XLSX)

S11 Table Table showing list and frequency of distribution of haplotypes recovered from the *G*. *f*. *fuscipes* samples mtDNA sequences analyzed from northern Uganda. (XLSX)
