# Supplementary figures and images for: Genetic diversity and population structure of the tsetse fly Glossina fuscipes fuscipes (Diptera: Glossinidae) in Northern Uganda: Implications for vector control
Source: PLoS Negl Trop Dis. 2017 Apr 28;11(4):e0005485. doi: 10.1371/journal.pntd.0005485 (PMC5425221; doi:10.1371/journal.pntd.0005485)

PCA eigenvalues

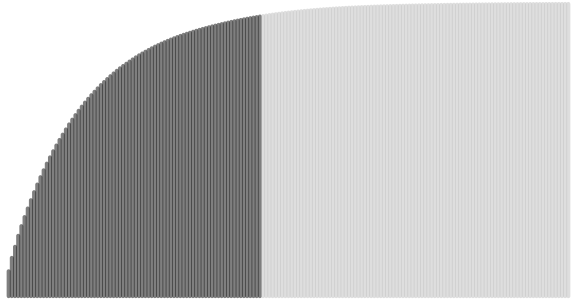

DA eigenvalues

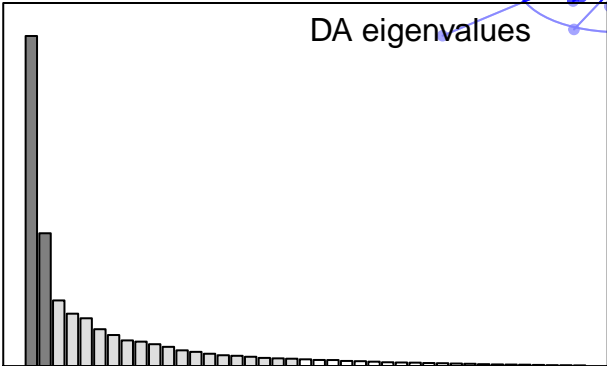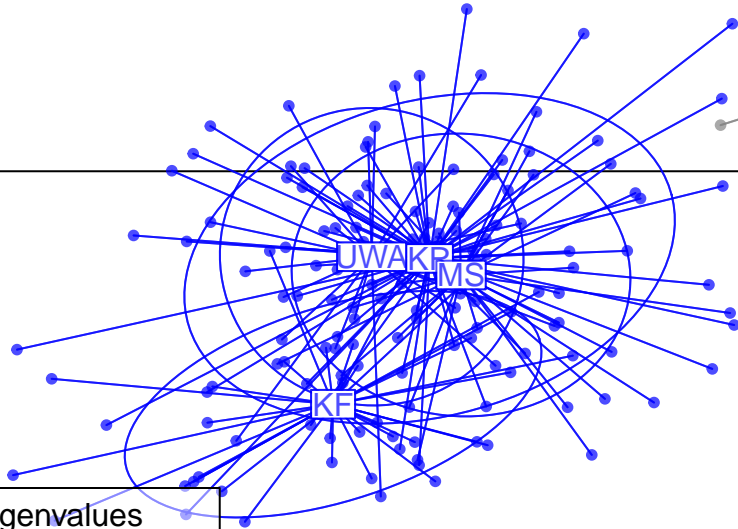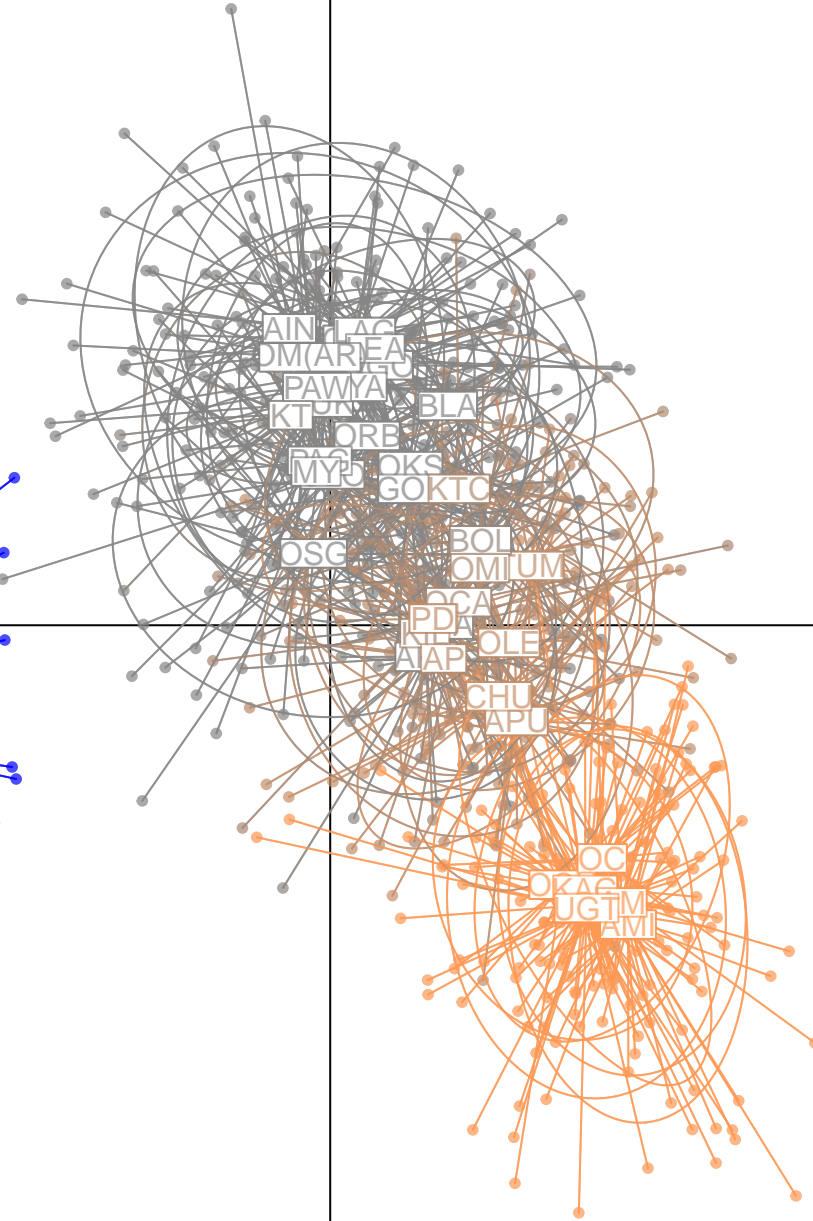

Supplement: S1 Fig — Two linear discriminants (LD1 and LD2) were used, following selection of principal components using a-score optimization, to plot G. f. fuscipes genotypes. Color codes are the same as in Fig 3. Letter codes represent sampling locations. Dots represent individual genotypes and the groups belonging to a sampling site as ellipses. Upper and bottom left insets show eigen values of principle components in relative magnitude. Black bars of eigen values show the proportion of principal components retained. (PDF) [file pntd.0005485.s001.pdf]

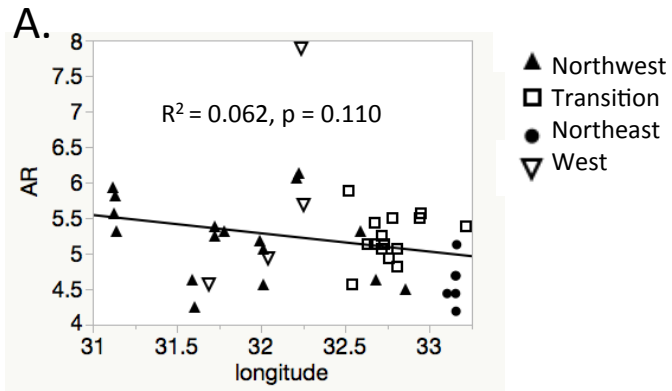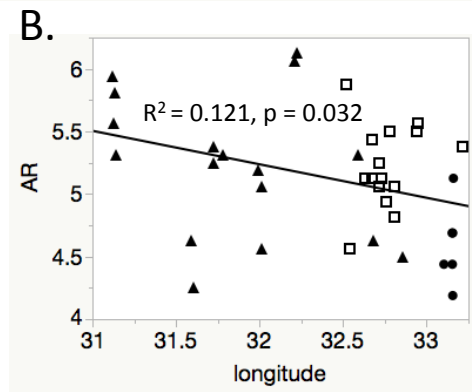

Supplement: S2 Fig — Triangles, diamonds, and squares identify sampling sites within the Northwest, Transition Zone, Northeast genetic units, respectively. (PDF) [file pntd.0005485.s002.pdf]
