# Supplementary material for: Genetic diversity and population structure of the tsetse fly Glossina fuscipes fuscipes (Diptera: Glossinidae) in Northern Uganda: Implications for vector control
Source: PLoS Negl Trop Dis. 2017 Apr 28;11(4):e0005485. doi: 10.1371/journal.pntd.0005485 (PMC5425221; doi:10.1371/journal.pntd.0005485)
Supplement: S2 Table — The table reports loci names followed by the forward (F) and reverse (R) primers names and sequences. The last column reports its source. M13 tails are marked with an asterisk (*). (DOCX) [file pntd.0005485.s004.docx]

**Supplementary material 2: Microsatellite loci names, primers names, their sequences and associated reference. F and R refer to forward and reverse primers, respectively. M13 tails are marked with an asterisk (*).**

| **Locus** | **Primer** | **Primer sequence** | **Source** |
| --- | --- | --- | --- |
| GmmA06 | GmA06F | ACTTCCATGTTATGTTCGTTGC | Hyseni *et al.,* 2011 |
|  | GmA06R | TGCCTTAGTTGAGAAACTCTGC |  |
| GmmB20 | GmmB20F | AAATGCATGTCTAACTGTCCG | Hyseni *et al.,* 2011 |
|  | GmmB20R | AGCAAAAGGCAACTAAAGTGATG |  |
| GmmD15 | GmmD15F | ACTGCATCTGCCTCTGTCG | Hyseni *et al.,* 2011 |
|  | GmmD15R | TGAACGAGAAAATGTGAATGGTAAG |  |
| GmmL03 | GmmL03F | ACAGTCCAATTTTCGCCCG | Hyseni *et al.,* 2011 |
|  | GmmL03R | GGCCAACAATGTCATAAACCG |  |
| GmmL11 | GmmL03F | CCACCACTAACAACGACAGC | Hyseni *et al.,* 2011 |
|  | GmmL03R | TGGCTGGTTACAAGATTGCAC |  |
| A03b | A03bF | AGGAATCAGTTAGTCTTCTGC | Brown *et al.,* 2008 |
|  | A03bR | ACTCGACCTCATCTATTCTG |  |
| B05 | B05F | CGCGCTTAGCTAGGAAACTC | Abila *et al.,* 2008 |
|  | B05R | AACGATTTGCTGTCCTCGAT |  |
| D05 | D05F | TTTCCTTCCAGACGAACTG | Abila *et al.,* 2008 |
|  | D05R | CTTGGTATGGTCGTACATGG |  |
| D101 | D101F | TGCCTTTACACTGCATACTACC | Abila *et al.,* 2008 |
|  | D101R | AAAAAGAGGAGCAATGATGTG |  |
| Gmm8 | Gmm8F | *CGCGCTTCAATGTTTGCTTTC | Baker *et al.,* 2001 |
|  | Gmm8R | TGCAGATGCAATGCGGAGAG |  |
| GpCAG29b | GpCAG29bF | *AACTATTGCTGGGCTCAC | Baker *et al.,* 2001 |
|  | GpCAG29b | AATTTCACTTCCACTCACCG |  |
| GpB20b | GpB20bF | *CAAAAGGGGAAAAGAAAGAAAGAGT | Ouma *et al.,* 2003 |
|  | GpB20bR | GTTTCGGCAGTAGATGGCAA |  |
| GpCAG133 | GpCAG133F | ATTTTTGCGTCAACGTGA | Baker *et al.,* 2001 |
|  | GpCAG133R | ATGAGGATGTTGTCCAGTTT |  |
| GpC10b | GpC10bF | *TTCAAGCACACTATTGCCAC | Ouma *et al.,* 2003 |
|  | GpC10bR | GCTGGCAAAGAAACTATTGA |  |
| Pg17 | Pg17F | *TGGCAAACTCTTCCATGTTT | Luna *et al.,* 2001 |
|  | Pg17R | GCTTACGTGAATCGTATCGAAT |  |
| Pg28 | Pg28F | *TCAAATTGTTCCCATCAAGGA | Luna *et al.,* 2001 |
|  | Pg28R | ATCGTTTTTAAAGGGTTTTAAGTTT |  |
