# Supplementary material for: Genetic diversity and population structure of the tsetse fly Glossina fuscipes fuscipes (Diptera: Glossinidae) in Northern Uganda: Implications for vector control
Source: PLoS Negl Trop Dis. 2017 Apr 28;11(4):e0005485. doi: 10.1371/journal.pntd.0005485 (PMC5425221; doi:10.1371/journal.pntd.0005485)
Supplement: S3 Table — (DOCX) [file pntd.0005485.s005.docx]

Supplementary Material 3: Polymorphism of loci tested

| **Loci** | A03b | B05 | C7b | CAG29 | GpCAG133 | D05 | D101 | Gmm8 | GpB20b | GpC10 | Pgp28 | GmL03 | GmmA06 | GmB20 | GmmL11 | GmD15 |
| --- | --- | --- | --- | --- | --- | --- | --- | --- | --- | --- | --- | --- | --- | --- | --- | --- |
| **Total Alleles** | 24 | 5 | 16 | 8 | 9 | 11 | 9 | 10 | 22 | 9 | 13 | 11 | 12 | 8 | 18 | 10 |
