# Supplementary material for: Genetic diversity and population structure of the tsetse fly Glossina fuscipes fuscipes (Diptera: Glossinidae) in Northern Uganda: Implications for vector control
Source: PLoS Negl Trop Dis. 2017 Apr 28;11(4):e0005485. doi: 10.1371/journal.pntd.0005485 (PMC5425221; doi:10.1371/journal.pntd.0005485)
Supplement: S7 Table — Results for the Northwest, Transition Zone, Northeast, West units, and all samples combined (Overall) are shown separately. Genetic group, root mean square values (R2), p-value for the Mantel test (p), and slope and intercept of the linear regressions are shown. Significant correlations are indicated in bold (p<0.05). (DOCX) [file pntd.0005485.s009.docx]

**Supplementary Material 7: Correlation statistics for tests of isolation by distance (IBD) using nuclear microsatellites based F_ST_ and mtDNA sequence based Φ_ST._**

| **Microsatellites** | | | | |
| --- | --- | --- | --- | --- |
| **Unit** | **R^2^** | **p** | **Slope** | **Intercept** |
| Northwest | 0.259 | **0.001** | 0.001 | 0.003 |
| Transition Zone | 0.216 | **0.001** | 0.001 | -0.005 |
| Northeast | 0.049 | 0.217 | 0.001 | -0.003 |
| West | 0.256 | 0.116 | 0.001 | 0.019 |
| Overall | 0.438 | **0.000** | 0.000 | 0.001 |
| **mtDNA** | | | | |
| **Unit** | **R^2^** | **p** | **Slope** | **Intercept** |
| Northwest | 0.425 | **0.001** | 0.004 | -0.152 |
| Transition Zone | 0.002 | 0.374 | 0.001 | -0.028 |
| Northeast | 0.014 | 0.672 | 0.013 | -0.041 |
| West | 0.001 | 0.366 | 0.003 | -0.072 |
| Overall | 0.490 | **0.001** | 0.000 | 0.000 |
